# *In Situ* Heart Isolation Featuring Closed Loop Recirculation: The Gold Standard for Optimum Cardiac Gene Transfer?

**DOI:** 10.4172/2379-1764.1000241

**Published:** 2017-10-06

**Authors:** Michael G Katz, Anthony S Fargnoli, Roger J Hajjar, Charles R Bridges

**Affiliations:** Cardiovascular Research Center, Department of Cardiology, Icahn School of Medicine at Mount Sinai, New York, USA

**Keywords:** Gene therapy, Molecular cardiac surgery with recirculating delivery, Cardiac isolation

## Abstract

The concept of delivering nucleic material encoding a therapeutic gene to the heart has arduously moved from hypothesis to a variety of high potential clinical applications. Despite the promise however, the results achieved have yet to be realized due to several problems that persist in the clinic. One of these identified problems is the need for an efficient delivery method which facilitates complete cardiotropism and minimizes collateral effects. Additional parameters impacting gene delivery that most need to be improved have been identified as follows: (1) Increasing the contact time of vector in coronary circulation permitting transfer, (2) Sustained intravascular flow rate and perfusion pressure to facilitate proper kinetics, (3) Modulation of cellular permeability to increase uptake efficiency, and once in the cells (4) Enhancing transcription and translation within the transfected cardiac cells, and (5) Obtaining the global gene distribution for maximum efficacy. Recently it was hypothesized that use of cardiopulmonary bypass may facilitate cardiac-selective gene transfer and permit vector delivery in the arrested heart in isolated “closed loop” recirculating model. This system was named molecular cardiac surgery with recirculating delivery (MCARD). The key components of this approach include: isolation of the heart from systemic organs, multiple pass recirculation of vector through the coronary vasculature, and removing the residual vector from the coronary circulation to minimize collateral expression. These attributes unique to a surgical approach such as MCARD can effectively increase vector transduction efficiency in coronary vasculature.

## Delivery Concepts for Successful Myocardial Gene Therapy

In the previous 25 years, there has been a substantial increase in the understanding of the aims of gene therapy, development of transgenic models of various diseases and synthetically packaging nucleic material in a variety of biologic therapeutics. Recently in parallel with clinical development, there have been numerous organized efforts to improve gene delivery techniques specifically tailored for cardiovascular gene therapies. Three major conclusions from previously published data are as follows: (1) Even the best engineered vectors such as those containing cardiac-specific promoter cannot limit the delivery of viral capsids to collateral organs or non-target tissue in the heart, (2) The route of administration of gene transfer is equally or more important than the vector or promoter system in larger species, and (3) Optimal gene transfer can be defined in terms of transfer ratio to the target organ versus inadvertent collateral transfer to evaluate efficiency. Thus, as additional cardiac gene therapies have moved into clinical trials, the drug delivery aspects specific to gene therapies have thrived opening and entirely new device field.

Despite significant interest and investment to optimize delivery however, an ideal cardiac gene delivery route of administration with complete cardiotropism and no collateral effects does not exist. Intravascular and intramyocardial delivery has been shown to limit transduction to the heart with varied results since systemic leakage is unavoidable given single pass gene transfer. Moreover, very rapid dilution featuring dramatically reduced vector concentration via larger circulating blood volume is typically observed. In addition, decreases in bioavailability are a problem as subsequent gene therapy products not reaching the heart are either absorbed by collateral organs or inactivated by the immune system. Thus, delivery systems addressing these concerns would offer substantial improvement for overall therapy with a key focus on several parameters.

## Key Parameters to Evaluate Various Cardiac Gene Delivery Methods

The gene therapy parameters impacting delivery that most need to be improved have been identified as follows: (1) Increasing the contact time of vector in coronary circulation permitting transfer, (2) Sustained intravascular flow rate and perfusion pressure to facilitate proper kinetics, (3) Modulation of cellular permeability to increase uptake efficiency, and once in the cells (4) Enhance transcription and translation within the transfected cardiac cells, and (5) Obtaining the global gene distribution for maximum efficacy.

## Technical Challenges for Clinically Relevant Cardiac Gene Delivery

Selective coronary catheterization with antegrade delivery was first tested in a rabbit model [1]. Today, it is the widely practiced technique utilized in clinical trials of heart failure gene therapy [2]. The benefits of this method include the possibility of repeated vector deliveries to the whole myocardium with homogenous gene expression and its safety record in providing a minimal invasive, high throughput system. However, the limited transduction and varied results with systemic leakage leading to significant collateral organ uptake led researchers to identify parameters influencing the efficiency of intracoronary transfer. If the gene delivery flow through the selective antegrade coronary administration is, for example, 20% of the flow rate of normal coronary blood flow, then only 20% of the vector infused would recirculate on the second pass through the system and only 0.8% would recirculate on the fourth pass. Thus, each virus would pass through the coronary circulation an average of <1.3 times or essentially one-pass kinetics. It is estimated that using simple selective antegrade coronary infusion technique, more than 99% of the vector disappears into the systemic circulation [3]. To address this obvious problem, there have been many new additional techniques to optimize selective antegrade coronary gene delivery including temporary interruption of coronary arterial flow [4], coronary venous blockade during gene transfer [5], changes of perfusion pressure and flow [6] and transient cardiac arrest and enhanced endothelial permeability with pharmacological agents [7]. However, all these methods were not clinically relevant due to their aggressive means in either manipulating anatomy or inflating devices in compromised high risk vessels.

The next advancement was selective intra-coronary retrograde delivery through the coronary sinus [8]. The use of this technique is undoubtedly a step forward compared with a selective antegrade intracoronary technique due to several factors. First, it can be much more easily applied in patients with diffuse atherosclerotic plaques in coronary arteries system. Second, it allows for prolonged adhesion time of the vector to the cardiac endothelium and overcomes the arterial resistance. Finally, it can reduce myocardial reperfusion injury [9]. Pressure regulated retrograde infusion into the anterior cardiac vein substantially increases gene expression in the targeted territory due to increased coronary passage time [10]. Another advantage is an enhancement of capillary filtration ratio in the venous part of the capillary bed and the ability to overcome the resistance of pre-capillary sphincters located before arterial capillaries [11]. Despite these advantages however, this method still cannot prevent the fast dilution of vector in the circulating blood with subsequent dissemination to the collateral organs.

## Molecular Cardiac Surgery Featuring Close-loop Recirculation System: A Case Study for Cardiac Surgical Gene Delivery Approaches

As a response to address the shortcomings of catheter based systems, researchers hypothesized that utilizing advanced device concepts leveraging consistent flow perfusion would provide a better means to increase bioavailability and reduce first pass effects. The ultimate device concept of this proposal is “closed-loop” recirculatory systems, which allowed separation of the coronary vascular bed from the systemic [12]. Recently it was hypothesized that use of cardiopulmonary bypass (CPB) may facilitate cardiac-selective gene transfer and allowing to make a vector delivery in stopped heart [13].

**Figure 1:**
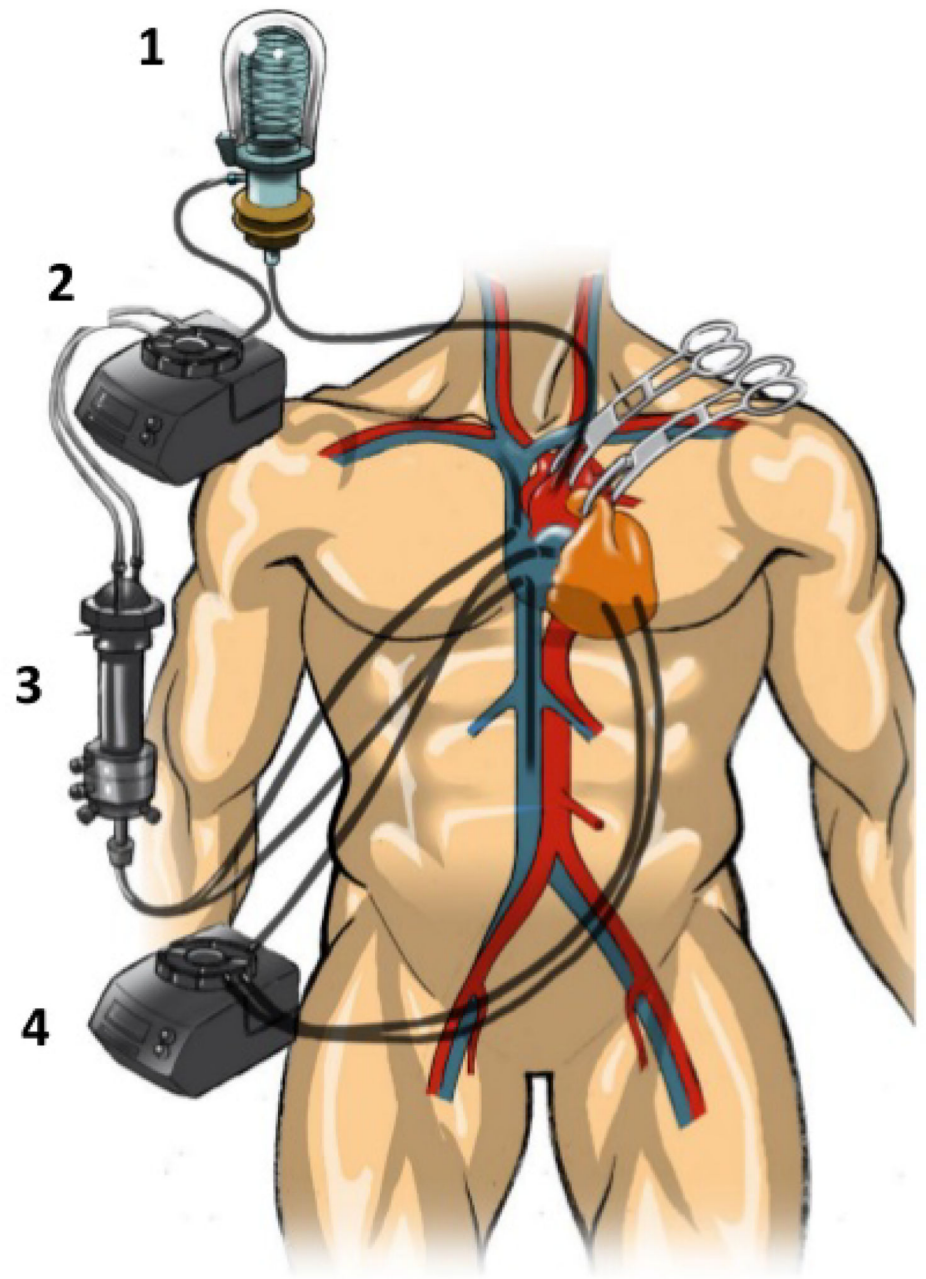
Cardiopulmonary bypass-based technology, e.g. molecular cardiac surgery with recirculating delivery (MCARD). After initiating systemic bypass, the vent cannulas were placed into left and right ventricles and connected to the venous limb of the circuit. The arterial limb is connected to the coronary sinus catheter. After stopping the heart with cardioplegia, recirculation commences for 20 min and then coronary circuit flashed. (1: Membrane oxygenator, 2: First roller pump for systemic circulation, 3: Heat exchanger, 4: Second roller pump for cardiac circulation).

Ultimately, one leading group in the field of cardiac gene delivery developed an isolated “closed loop” recirculating model. This system was named molecular cardiac surgery with recirculating delivery (MCARD) and demonstrated effectiveness in the large animal permitting vector recirculation for 20 min [14]. The key components of this approach include isolation of the heart from the other organs, multiple recirculation of vector through the coronary vasculature and washing the residual vector from the coronary circulation to minimize collateral expression. Essentially, the “closed-loop” recirculation method leverages two separate, oxygenated circuits for the heart and systemic circulations (Figure 1). A key advantage unique to this system is the control of perfusion flow and pressures permitting multiple passes of vector through the heart. This in theory and for practical purposes optimizes of a number of variables that have been shown to be important determinants of the efficiency of intravascular cardiac gene delivery. In addition despite its invasiveness as compared with other systems, this method is clinically translatable and could be used as an adjunct treatment to other cardiac surgical procedures where cardiopulmonary bypass is utilized provided a given treatment would justify a favorable benefit over the long term [15].

## Conclusion and Future Perspective

Summarizing, it has been demonstrated that complete surgical isolation of the heart *in situ*, using CPB with high-pressure retrograde coronary sinus infusion with multiple-pass recirculation of vector through the heart results in an increase of several orders of magnitude in cardiac marker gene activities compared with controls, receiving retrograde infusion of adenovirus without CPB and without cardiac isolation [16]. The principal strength of this technology includes a dramatic (>1000-fold) increase in transduction efficiency, the extension of vector residence time, the ability to manipulate endothelial permeability, the avoidance of an immune response to the vector and the ability to washout the vector post gene delivery limiting collateral organ exposure [17]. “Closed loop” systems such as MCARD can provide robust gene expression with no detectable extra-cardiac transfer and no detrimental effects on major organ function. Despite these advantages however MCARD’s position in terms of applicability would only be for select adjunctive cardiac surgery procedures and not standalone due to its invasiveness. Therefore, a new conceived unaddressed market is adapting minimally invasive robotic surgery tools for delivery purposes.

Minimally invasive cardiac surgical and or image guided interventional techniques and tools would serve to broaden the base of potential patients that would benefit from cardiac gene therapy. Currently, there is a revolution in cardiac surgery featuring advanced robotic applications that access the heart with minimal incision and access points. Additionally, minimally invasive grafts, valve replacements and other supporting devices would offer a means to integrate “gene eluting” or other inspired combination devices applied in patients.
